# Artificial intelligence and guidance of medicine in the bubble

**DOI:** 10.1186/s13578-021-00623-3

**Published:** 2021-06-09

**Authors:** Asma Akbar, Nagavalli Pillalamarri, Sriya Jonnakuti, Mujib Ullah

**Affiliations:** 1 Institute for Immunity and Transplantation, Stem Cell Biology and Regenerative Medicine, School of Medicine, Stanford University, Palo Alto, CA 94304 USA; 2 Molecular Medicine, Department of Biomedical Innovation and Bioengineering, School of Medicine, Stanford University, Palo Alto, CA USA

**Keywords:** Artificial intelligence, Microbubbles, Nano-vesicles, Drug transportation, Targeted therapies

## Abstract

Microbubbles are nanosized gas-filled bubbles. They are used in clinical diagnostics, in medical imaging, as contrast agents in ultrasound imaging, and as transporters for targeted drug delivery. They can also be used to treat thrombosis, neoplastic diseases, open arteries and vascular plaques and for localized transport of chemotherapies in cancer patients. Microbubbles can be filled with any type of therapeutics, cure agents, growth factors, extracellular vesicles, exosomes, miRNAs, and drugs. Microbubbles protect their cargo from immune attack because of their specialized encapsulated shell composed of lipid and protein. Filled with curative medicine, they could effectively circulate through the whole body safely and efficiently to reach the target area. The advanced bubble-based drug-delivery system, integrated with artificial intelligence for guidance, holds great promise for the targeted delivery of drugs and medicines.

## Introduction

Many drugs and medicines are susceptible to degradation, which makes it challenging to formulate them and deliver them to specific targets [1, 2]. The chemistry of these drugs makes the process even more challenging because it can lead to nonspecific side effects, interrupt normal physiology of intracellular receptors, damage healthy tissues, and result in unguided delivery [2–4]. Additionally, these drugs have reduced permeation across biological barriers, affinity towards unspecific sites, and a tendency to unload chemicals to multiple healthy targets [3]. To overcome these shortcomings, microbubbles deliver their cargo to molecular sites of disease while being tracked in real time by the latest simulation of artificial intelligence [3, 5, 6].

AI could potentially enhance the effectiveness of microbubble technology [6–8]. Bubbles guided by AI, and the medicine they encapsulate, have the power to improve the visualization of cardiac disease, which will give new life to the field of echocardiography or focused ultrasound imaging of the heart [9–12]. They are routinely used to evaluate myocardial perfusion and heart function and in kidney dialysis [11, 12]. Clinically, microbubbles are established for routine screening of a range of diseases, including cancer, cancerous lesions, inflammatory processes, cardiovascular pathologies, and diseases associated with aging, Table 1 [12–15].

**Table 1:** Role of microbubbles in different diseases and in different clinical trials

S. No	Name	Type	Disease type	Role	Technology	*Clinical trials*
1	The Role of Different Imaging Methods in the Diagnosis of Gallbladder Polyps	Interventional	Polyp of Gallbladder	Preoperative differential diagnostic accuracies of gallbladder polypoid lesions	Device: Abdominal high-resolution CT Device: Contrast-enhanced ultrasonography Procedure: Cholecystectomy	*NCT02762227*
2	Novel MRI-Guided Ultrasound Stimulated Microbubble Radiation Treatment for Patients with Chest-wall and Locally-Advanced Breast Cancer	Interventional	Breast Cancer	Novel MRI-guided ultrasound stimulated microbubble treatment enhances radiation effects in humans receiving external beam radiotherapy delivered using radiation therapy device	Patients with locally advanced breast cancer and chest wall tumours will receive MRI-guided ultrasound-stimulated microbubble-treatment combined with radiotherapy	*NCT04431674*
3	The Use of Focused Ultrasound and Microbubble Infusion for Altering Brain Perfusion and the Blood Brain Barrier	Interventional	Low Grade Glioma of Brain	The ability of focused ultrasound combined with microbubbles to open the blood brain barrier has the potential to revolutionize the delivery of therapeutic agents to the brain, allowing for more localized and efficient delivery	The ultrasound treatment will last either 1 h or 20 min total time for the DWL device. An infusion of Definity microbubbles will be infused intravenously over ten to thirty minutes as per routine approved application. Definity will be 1.3 mL added to 50 mL saline and infused no faster than 4 mL/minute	*NCT04063514*
4	Microbubbles and Ultrasound in Stroke Trial	Interventional	Acute Ischemic Stroke	Transcranial 2-MHz ultrasound combined with intravenous administration of microbubbles improves early recanalization in patients with acute ischemic stroke caused by middle cerebral artery proximal occlusion treated with intravenous alteplase within 3 h of symptom onset	Radiation: Ultrasound 2-MHz, low intensity transcranial ultrasound Drug: Levovist D-Galactose and palmitic palmitique intravenous 4 g	*NCT00222040*
5	Microbubble Cavitation for Improving Hepatocellular Carcinoma Radioembolization	Interventional	Hepatocellular Carcinoma Liver Cancer	Localized microbubble cavitation triggered by noninvasive ultrasound has been shown to sensitize malignant tissue to radiotherapy by inducing vascular endothelial cell apoptosis	Patients receive perflutren protein-type A microspheres IV over 10 min and undergo CEUS over 60 min at 1–6 h post radioembolization and at approximately 7 and 14 days after yttrium Y-90 radioembolization	*NCT03199274*
6	Targeted Delivery of Chemotherapy with Ultrasound and Microbubbles (SONCHIMIO)	Interventional	Colorectal Cancer Hepatic Metastases	The oscillations of ultrasound (US) contrast agent microbubbles under their activation by US waves engender a modulation of the permeability of biological barriers amplifying hence the extravasation of drugs/fluorescent markers through a process known as sonoporation	Liver metastases randomized to receive sonoporation (US waves + gaseous microbubbles). The patient continues to receive the usual systemic chemotherapy	*NCT03458975*

Molecular imaging is an advanced way for decoding the biological processes to visualize and reveal the cellular events at molecular level [16, 17]. For example, quantum dots are photostable for longer duration and enhance the imaging of deep tissues [17]. Similarly, the fluorescence imaging with indocyanine green based system is valuable for monitoring of surgical procedures [17, 18]. Magnetic resonance imaging is clinically applied to visualize and expose the structural and pathological changes [19, 20]. Combination of different nanoparticles and contrast agents makes the field of molecular imaging more appealing for clinical applications [21–23]. The process of molecular imaging can be clinically improved by designing and engineering of new class of contrast agents, which are more sensitive, targeted, non-toxic and precise for molecular identification [24–26]. Such as monoclonal antibodies, nanoprobes, quantum dots, molecular dyes, and other targeting signatures are routinely attached to the surface of microbubble and extracellular vesicles for clinical monitoring of treatment process [5, 14, 21, 22, 26, 27]. Therapeutic drugs and medicines include growth factors, antibodies, peptides and recombinant proteins; microbubbles increase the effectiveness, specificity, and potency of these therapeutics [28–30].

## The role of guided microbubbles in different diseases

**Fig. 1:**
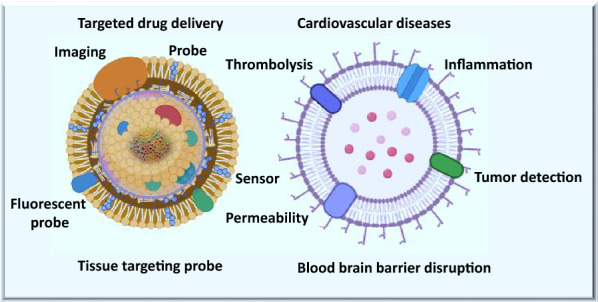
Role of guided microbubbles in drug delivery and imaging and in different diseases

Conventional medicine are known for their shortcomings such as toxicity to healthy tissues, not very specific to the targets, do not have the ability to cross the blood brain barrier, and have other side effects which restrict their applications [9, 31, 32]. New technologies have significantly improved the perspective of precision medicine [33–35]. The smart approach of using microbubbles loaded with degradable loaded drugs have the potential to deliver the medicine precisely to the targets where it is needed [36, 37]. Precision medicine (such as guided microbubbles) use the high throughput knowledge and artificial intelligence to enhance the process of clinical diagnosis and treatment [38, 39]. Moreover, the precision approach can also be used for preventive and surveillance measures of diseases. treatment efficiency and real time monitoring of drugs [39, 40]. Microbubbles loaded with drug are guided carriers that deliver medicine to targeted sites [2, 30, 41]. These bubbles increase the localized drug concentration at the site of disease, and mimicking the toxicity and unwanted delivery to healthy tissues and surrounding microenvironment [41, 42]. Ultrasound-guided microbubbles are routinely used in the treatment of many diseases’ such as cardiovascular disorders including thrombolysis, in the clinical imaging of tumor sites, and diagnosis of cancer and therapeutics [42–44]. Studies have shown that microbubbles loaded with drugs (such as growth factors, precision medicine, tissue plasminogen activator (tPA), regenerative molecules, and imaging probes) have improved the clinical outcome in different diseases [45, 46]. Microbubbles loaded with tPA successfully dissolved blood clots precisely at the tissue target sites, and bubbles loaded with regenerative cargo improved the healing effects in tissues repair process [42–44, 47]. Technology-guided microbubbles are well studied for targeted release of drugs at inflammatory and tumor sites Fig. 1 [3, 48]. Monoclonal antibodies, cytokines, tumor inhibitors, chimeric antigen receptor T cells therapies, and clinical chemotherapeutic drugs (for example, 5-fluorouracil and doxycycline) have successfully been loaded into microbubbles and applied in the treatment of neck, breast, pancreatic, ovarian, and hepatocellular carcinoma [49–51]. Ultrasound-targeted microbubbles have already been validated as an effective method for delivering microRNAs to tumor sites in clinical treatment of human malignancies [38, 49].

**Fig. 2:**
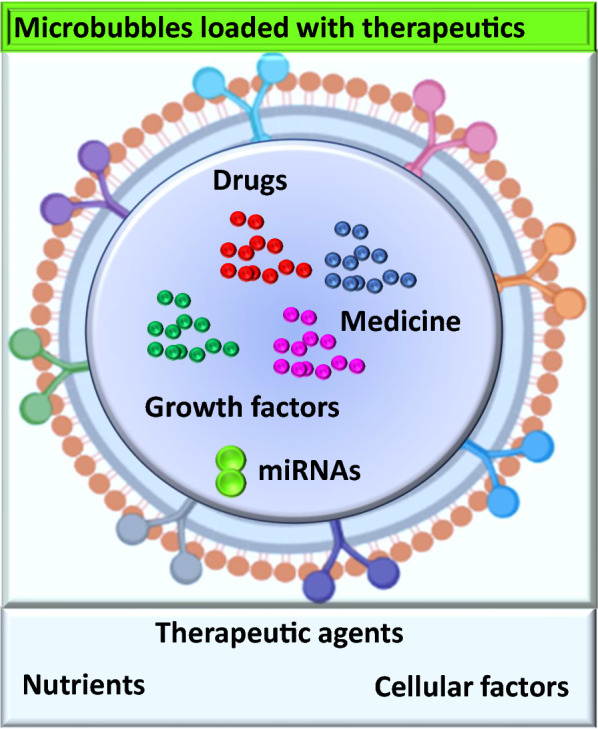
Microbubbles and loaded therapeutic cargo

**Fig. 3:**
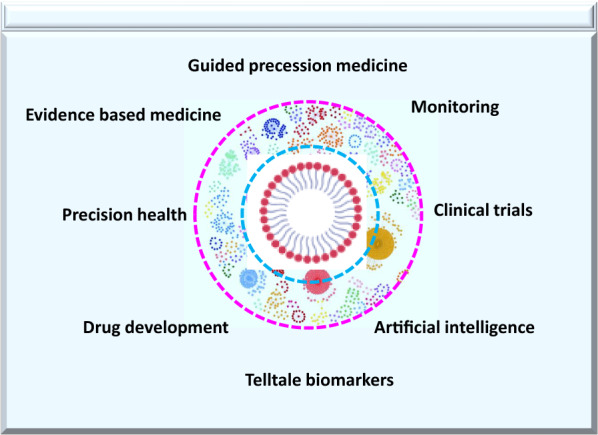
Guided microbubbles and technology

AI could be used for medical imaging, patient monitoring, and for the targeted release of drugs at the damaged sites. It will enhance the therapeutic efficiency by increasing the localized drug concentration at diseased sites [8]. Microbubbles can cross the blood–brain barrier, meaning drugs can reach any brain cell in a targeted manner [8]. Microbubbles loaded with glial cell-derived neurotrophic factor (GDNF) and brain-derived neurotrophic factor (BDNF) have been shown to specifically accelerate the cell survival of dopaminergic neurons and protect neurons in treatment of many diseases, such as stroke, Alzheimer’s, Parkinson’s disease, seizure disorders, brain or spinal injuries, and other neurological disorders [8, 15, 44, 52–54]. Ultrasound has the potential for activation of drug release at targeted regions, and has the ability of precise-imaging to identify the diseased sites, thus enhancing the implications of microbubbles in treatment of different diseases [44, 55]. This technology includes the potential to monitor the drug and treatment response in real time, which increases the effectiveness of this approach Figs. 1, 2 and 3.

## Technology and microbubbles

The technology behind the smart design of microbubbles has attracted great attention due to its wide application in many fields of science and technology [21, 56]. The nano-sized microbubbles are negatively charged [56, 57]. Particles with positive charges, known as free radicals, engulf electrons from healthy cells to neutralize their own charge, causing cellular damage [15, 58, 59]. In contrast, negatively charged bubbles fight free radicals to improve the health of damaged cells and detoxify the inflammatory fluids in diseased tissue [15, 60].

Microbubbles newly designed through biomedical engineering and nanomaterials approaches are crucial for intracellular delivery of proteins, drugs, growth factors, and peptides, Fig. 2 [9, 30, 42]. They may revolutionize the whole biopharmaceutical drug industry [14, 58]. Although technological breakthroughs have been made in the development, monitoring, and tracking of drugs by artificial intelligence and in the delivery of biopharmaceutical drugs, challenges and unanswered questions remain [61]. The medicine in the bubbles can target both extracellular and intracellular targets and guide the localized drug delivery to specific sites [61].

## Medicine and the machine

Artificial intelligence (AI)–based technologies have the potential to transform the healthcare industry by deriving innovative approaches to the discovery of drugs, Fig. 3 [5, 61]. Examples of innovation through AI range from self-driving cars to pattern- and image-recognition tools to clinical diagnostics that allow expedited drug discovery, earlier detection of disease, more precise diagnosis, identification of new biomarkers, and development of personalized diagnostics and therapeutics [6, 7, 10, 62]. AI has the power to treat, diagnose, cure, mitigate, or prevent disease or other critical or serious conditions [29, 61, 63]. Recent studies have shown that AI can expedite diabetic retinopathy and eye scan [29, 61, 63, 64]. AI has incredible pattern-recognizing abilities within big data and thus holds the potential to solve many key clinical challenges [64]. Leveraging AI with microbubble technology may expedite and enhance early detection of disease and patient care [5, 62].

## Medicine in microbubbles and the blood–brain barrier

The blood–brain barrier (BBB) is responsible for protection against circulating toxins, preventing harmful pathogens from entering the brain [44, 65]. The defensive wall of the BBB prevents brain infections, but it also blocks medicines that could treat brain diseases, neurological disorders, and neurodegenerative diseases [44, 65, 66]. This protective wall presents an obstacle for most of the available drugs in the market [66, 67]. Medicine in microbubbles, in contrast, can reach and open the BBB to target the disease site effectively instead of circulating randomly in the system [67, 68]. The brain is the only organ known to have its own security system; however, medicine in the bubbles breaks the defensive wall of the BBB and allows lifesaving drugs to reach their targets to repair the injured or diseased brain [68]. Usually drugs are chemicals, and the brain senses these harsh molecules and blocks its defensive door using the BBB; however, medicines in bubbles are difficult to interpret as chemicals or dangerous enemies as they are encapsulated in a shell, Figs. 1, 2 and 3 [15, 53, 66, 68].

## Conclusion

Microbubbles have the potential to protect their cargo from degradation, restrict the drug release to disease sites, and prevent nonspecific drug delivery to healthy tissues [69, 70]. Medicine in bubbles enhances targeted drug delivery, tumor targeting, ultrasound imaging, and intracellular drug release [7, 71]. These microbubbles can be used for the delivery of oxygen in stroke patients, and delivery of immune cells in those patients who has weak immune system [54, 72, 73]. AI-powered capabilities, including data integration and interpretation, are fundamental for clinical transformation of microbubbles to enhance treatment efficacy [7, 38]. Leveraging technology will enhance the ability of microbubbles and extracellular vesicles for oxygen release to energizes cells and stimulates the immune system against different diseases [74, 75]. Ultrasound guided microbubbles can be used for opening blocked arteries, for increasing the permeability of blood brain barrier and drug delivery to those tissues which are otherwise difficult target for conventional drug delivery [76–78].

The delivery of medicine in bubbles has some limitations, such as undesired shell cracking due to acoustic pressure, limited capacity for drug loading and cavitation in the ultrasound field [30, 61]. The biostability of microbubbles is poor in some organs, less biocompatible, structurally unstable, and limited circulation time in certain tissues. Sometimes these bubbles have difficulty reaching deep and hard ossified tissues [30, 38]. The safety, ethics, effectiveness, and functionality of the process should be considered to improve the development of next generation of microbubbles with innovative engineering approaches to enhance the drug loading capacity of bubbles [21, 79–81]. Designing of bubbles for precise imaging should revolutionize the field of molecular imaging and precision medicine for treatment of cancer, aging, cardiovascular and neurological diseases [13, 30, 82].

Microbubbles are inert, nonreactive vesicles which makes them ideal for molecular imaging, bypassing microcirculation, and ideal cargo for conventionally challenged targets [83–85]. Although there are still some challenges in the clinical translation, but scientists are expecting that newly designed microbubbles can be leveraged to AI methods and techniques [86, 87]. These emerging bubbles can be used to train neural networks and other tissues and to monitor drugs for real time imaging and precise treatment [87–89]. Microbubbles with integrated molecular sensor-probes have the ability to distinguish contrast agents and differentiate healthy versus diseased sites [90, 91]. The technological platform of microbubbles applications should be upgraded with AI integration for more safer, and real-time tracking of drugs in clinical translation [92]. Further research of guided microbubbles is needed to explore the field drug delivery. The field of bioengineering for designing smart microbubbles will revolutionize this technology further, which has already been shown its role in real time molecular imaging and precise treatment of different diseases such as cardiovascular and neurological disorders [93, 94]. The integration of AI and other technologies in the field of microbubbles will accelerate the development of strategies for detection, prevention, diagnosis, treatment, and cure.

## Data Availability

Not applicable.

## Abbreviations

EVs: Extracellular vesicles
BDNF: Brain-derived neurotrophic factor
GDNF: Glial cell line-derived neurotrophic factor
BBB: Blood–brain barrier
